# Heart rate responses to standardized trauma-related pictures in acute posttraumatic stress disorder

**DOI:** 10.1016/j.ijpsycho.2010.04.009

**Published:** 2010-10

**Authors:** Anke Ehlers, Oliver Suendermann, Inga Boellinghaus, Anna Vossbeck-Elsebusch, Matthias Gamer, Emma Briddon, Melanie Walwyn Martin, Edward Glucksman

**Affiliations:** a King's College London, Institute of Psychiatry, Department of Psychology, London, UK; b University Medical Center Hamburg-Eppendorf, Department of Systems Neuroscience, Hamburg, Germany; c King's College Hospital, Emergency Department, London, UK

**Keywords:** Posttraumatic stress disorder, Depression, Heart rate, Associative learning, Anxiety, Cognitive models, Stimulus generalization

## Abstract

Physiological responses to trauma reminders are one of the core symptoms of posttraumatic stress disorder (PTSD). Nevertheless, screening measures for PTSD largely rely on symptom self-reports. It has been suggested that psychophysiological assessments may be useful in identifying trauma survivors with PTSD (Orr and Roth, 2000). This study investigated whether heart rate (HR) responses to standardized trauma-related pictures distinguish between trauma survivors with and without acute PTSD.

Survivors of motor vehicle accidents or physical assaults (*N* = 162) watched standardized trauma-related, generally threatening and neutral pictures at 1 month post-trauma while their ECG was recorded. At 1 and 6 months, structured clinical interviews assessed PTSD diagnoses. Participants completed self-report measures of PTSD severity and depression, peritraumatic responses, coping behaviors and appraisals.

Trauma survivors with acute PTSD showed greater HR responses to trauma-related pictures than those without PTSD, as indicated by a less pronounced mean deceleration, greater peak responses, and a greater proportion showing HR acceleration of greater than 1 beat per minute. There were no group differences in HR responses to generally threatening or neutral pictures. HR responses to trauma-related pictures contributed to the prediction of PTSD diagnosis over and above what could be predicted from self-reports of PTSD and depression. HR responses to trauma-related pictures were related to fear and data-driven processing during the trauma, safety behaviors, suppression of trauma memories, and overgeneralized appraisals of danger.

The results suggest that HR responses to standardized trauma-related pictures may help identify a subgroup of patients with acute PTSD who show generalized fear responses to trauma reminders. The early generalization of triggers of reexperiencing symptoms observed in this study is consistent with associative learning and cognitive models of PTSD.

## 1 Introduction

After traumatic events, most people develop some post-trauma symptoms such as intrusive memories or being upset by reminders of the event. However, the majority of trauma survivors recover on their own and only a minority develop chronic posttraumatic stress disorder (PTSD) (Kessler et al., 1995; Rothbaum et al., 1992). The question of how best to identify trauma survivors who will not recover is of theoretical and practical interest. Current screening for PTSD relies largely on self-report symptom measures (Brewin, 2005). However, there is evidence that self-reported symptoms lead to an overestimation of PTSD risk (e.g. Engelhard et al., 2007).

This raises the question of whether psychophysiological responses may help in identifying people with PTSD (Orr and Roth, 2000). Wilhelm and Roth (2001) argued that physiological assessment in anxiety disorders such as PTSD may be clinically useful in three ways, (1) quantifying specific symptoms of anxiety disorders, (2) helping understand the underlying mechanisms, and (3) providing evidence for subtypes of the disorder.

Physiological responses to trauma reminders are among the core symptoms of PTSD (American Psychiatric Association, 1994) and have been shown to be highly specific to trauma survivors with PTSD (e.g. Ehlers et al., 1998; Ehring et al., 2007). This raises the question of whether the precision of PTSD screening can be enhanced by objective assessment of physiological responses to trauma reminders. Orr and Roth (2000) suggested that “… psychophysiological assessment of responsivity to trauma-related cues may be informative regarding the general presence, absence, or severity of PTSD” (p. 231). Like self-reports, physiological responses to trauma reminders may be particular helpful in increasing the specificity of the prediction of PTSD in that they may help identify people who report high scores on questionnaires for reasons other than PTSD. For example, Orr and Roth found that physiological responses to script-driven imagery of the trauma differentiated between participants with and without PTSD in a series of studies with a sensitivity of 60% and a specificity of 89%.

A recent meta-analysis (Pole, 2007) identified 17 studies of psychophysiological responses to standardized trauma cues in PTSD, and 22 studies using idiographic trauma cues. These studies used a range of measures including heart rate (HR), skin conductance, EMG or blood pressure. Responses to standardized cues across these measures identified PTSD with a mean sensitivity of 77% and a mean specificity of 91%; and responses to idiographic trauma cues identified PTSD with a mean sensitivity of 65% and mean specificity of 83%. An open question is whether the satisfactory sensitivity and specificity observed in these studies also applies to the *early* identification of trauma survivors with PTSD. Most of the studies reviewed by Pole (2007) were cross-sectional comparisons of survivors of very distant traumas, such as combat or sexual abuse in childhood (e.g. Donagh-Coyle et al., 2001, Orr et al., 1993). This limits the conclusiveness of the results for acute PTSD, as the heightened physiological reactivity may be a consequence of chronic PTSD rather than a factor contributing to its development.

Few studies to date have investigated physiological responses to trauma reminders soon after trauma. Two studies suggest that trauma survivors with acute PTSD show greater heart rate (HR) responses to trauma reminders than those without PTSD. Elsesser et al. (2004) found that chronic PTSD patients and recent trauma survivors who met criteria for acute stress disorders (ASD) at 6 weeks after the trauma showed heart rate acceleration to individualized trauma-related pictures, whereas nontraumatized controls and survivors without ASD showed heart rate deceleration. The groups did not differ in HR responses to generally threatening or neutral pictures. In a follow-up study of the recent trauma survivors from the Elsesser et al. (2004) study, Elsesser et al. (2005) further found that greater HR responses to the individualized trauma-related pictures predicted PTSD symptoms 3 months later. Blanchard et al. (1996) studied survivors of motor vehicle accidents (MVA) at about 2.5 months after the trauma and found that greater HR responses to audiotaped individualized scripts describing the participants' accident, but not responses to other stressors, distinguished survivors with PTSD from those without PTSD and nontraumatized controls. Furthermore, HR acceleration to individualized MVA scripts predicted chronicity of PTSD at 1 year in 48 participants who had PTSD at the initial assessment.

These studies suggest that early psychophysiological responses may indeed be useful in identifying trauma survivors with acute PTSD. Both studies used *idiographi*c trauma reminders: script-driven-imagery of the trauma (Blanchard et al., 1996) or idiographic pictures selected for their personal relevance to each participant (Elsesser et al., 2004). It is currently unclear whether HR responses to *standardized* trauma reminders can be used to identify people with PTSD soon after trauma, which would increase the ease of application for screening purposes.

There are theoretical reasons to expect greater HR responses to standardized trauma reminders in survivors with PTSD than those without PTSD. Associative learning models of PTSD suggest that during trauma fear responses become associated with stimuli present at the time (e.g. Foa et al., 1989; Keane et al., 1985). The HR responses to idiographic trauma reminders observed in the Elsesser et al. (2004) and Blanchard et al. (1996) studies are consistent with these models and resemble the responses of phobic patients to their phobic stimuli (Sartory, 1983). Associative learning models of PTSD also suggest that fear responses generalize more broadly to stimuli and situations that resemble the original trauma. Thus, one would expect trauma survivors with PTSD to show stimulus generalization soon after the trauma, and to respond to standardized trauma reminders with a phobic response pattern.

However, the results on early physiological responses to standardized trauma cues are mixed so far. One study of responses to an accident-related picture in motor vehicle accident (MVA) survivors reported positive findings (Rabe et al., 2006), but Blanchard et al. (1994, 1996) did not find that responses to guided imagery of a standard MVA script or videotapes of car crashes distinguished between survivors with and without PTSD.

The present prospective study was designed to investigate whether HR responses to standardized trauma-related pictures distinguish between trauma survivors with and without acute PTSD at 1 month post-trauma. HR responses were chosen because Pole's (2007) meta-analysis suggested that these are particularly sensitive in distinguishing between trauma survivors with and without PTSD. Similarly, HR response was the only physiological response measure that distinguished between trauma survivors with and without acute PTSD in Blanchard et al.'s (1996) and Elsesser et al.'s (2004) studies. The present study recruited trauma survivors soon after the trauma, and assessed PTSD diagnoses and self-reports of PTSD symptoms and depression at 1 and 6 months. Depression measures were included as depression has been shown to be a predictor of chronic PTSD (e.g. Shalev et al., 1998).

Finally, the present study examined correlates of HR responses of theoretical interest. Associative learning models of PTSD would predict an association between HR responses and degree of fear during the trauma, as the strength of conditioned responses depends on the strength of the unconditioned response (Keane et al., 1985; Foa et al., 1989). If HR responses to trauma-related pictures are an indicator of learned fear responses, one would also expect them to be related to phobic behaviors, such as safety-seeking behaviors that people with PTSD use to prevent future harm (Dunmore et al., 2001; Ehring et al., 2008). Furthermore, cognitive models of PTSD would predict a relationship between HR responses and perceptual processing during the trauma. Perceptual processing is thought to facilitate memory processes that lead to easy triggering of reexperiencing symptoms, which include physiological responses to trauma reminders such as increases in heart rate (Brewin et al., 1996; Ehlers and Clark, 2000; Ehlers et al., 2004). Furthermore, cognitive models of PTSD would predict a relationship between distressing reexperiencing symptoms (and thus HR responses to trauma reminders) and efforts to suppress trauma memories (Ehlers and Steil, 1995; Ehlers and Clark, 2000). Effortful suppression of trauma memories has been shown to predict PTSD (Ehlers et al., 1998; Ehring et al., 2008). Cognitive models (Ehlers and Clark, 2000) would further suggest specificity in the relationship between HR responses and post-trauma beliefs. HR responses would be expected to correlate with beliefs that the world is dangerous and the individual is at risk of further trauma. However, beliefs about internal threat such as negative beliefs about the self (e.g., ‘I am inadequate’, ‘I am weak’) would not be expected to correlate with HR responses to trauma-related pictures.

In sum, the present study investigated the following hypotheses:

Hypothesis 1 Participants with acute PTSD at 1 month after the trauma show greater HR responses to standardized trauma-related pictures than participants without PTSD, but not to generally threatening or neutral pictures.

Hypothesis 2 HR responses to trauma-related pictures add to the prediction of acute PTSD over and above what can be predicted from self-reported symptoms.

Hypothesis 3 HR responses to trauma-related pictures predict PTSD symptom severity at 6 months.

Hypothesis 4 HR responses to trauma-related pictures are related to fear and perceptual processing during the trauma. They are also related to dysfunctional coping strategies, namely safety-seeking behaviors and efforts to suppress trauma memories. They are related to negative beliefs about the dangerousness of the world, but not to negative appraisals of the self.

## 2 Materials and methods

### 2.1 Participants

Participants were recruited from assault or motor vehicle accident (MVA) survivors who were treated for their injuries at the Emergency Department of a large urban teaching hospital during the period of November 2004 to March 2006. To be eligible for the study, participants had to meet the trauma (A) criterion of DSM-IV (American Psychiatric Association, 1994), and understand and speak English fluently enough to be able to answer interview questions and fill in questionnaires. Participants with current psychosis and substance dependence, as well as those who could not remember the event (e.g., due to a head injury) were excluded. A total of 199 trauma survivors were recruited shortly after their trauma and attended the research session. For 18 of these, HR data were missing because of technical problems or because the participant did not have enough time. Three other participants had to leave the session early and thus had missing data for the diagnostic interview (missing PTSD diagnosis). A further 16 had to be excluded prior to analysis for the following reasons: use of medication that may affect HR such as β-blockers or tricyclic antidepressants (*n* = 5); intoxication with alcohol or drugs at the time of the trauma (*n* = 2); problematic recording, e.g., irregular heart rate with frequent extra systoles or breathing artefacts (*n* = 8); forgot to bring glasses (*n* = 1). Therefore, the final sample size comprised 162 trauma survivors (68 assault survivors and 94 MVA survivors). Of these, 66 (40.7%) met the criteria for acute PTSD at 1 month after the trauma (we will refer to these participants as the PTSD group) according to the *Structured Clinical Interview for DSM-IV* (SCID) (First et al., 1996), and 96 (59.3%) did not have PTSD at 1 month (no PTSD group).

Ninety one percent (*n* = 148) of the participants took part in the 6-month follow-up, and 25 (16.9 %) of these still met PTSD diagnostic criteria at follow-up. If participants had PTSD at follow-up (or earlier if the participant was very distressed or at risk), treatment was arranged via their family doctors. All participants were asked whether they had received any treatment for PTSD at the 6-month follow-up. Four participants reported that they had received an effective treatment for PTSD (psychological treatment or medication); their pretreatment scores were used for data analysis.

### 2.2 Material

Participants viewed a series of pictures; 15 pictures were related to the participants' trauma (i.e., assault survivors saw assault-related pictures such as a person being attacked by a gang; and MVA survivors saw accident-related pictures such as a crashed car), 10 generally threatening pictures (e.g. a collapsing building), and 10 neutral pictures (e.g. TV and book) in pseudorandom order. Pictures were mainly drawn from the International Affective Picture System (Lang et al., 2005) and from the pictures used by Elsesser et al. (2004). Details of the pictures are shown in the appendix. Pictures of the same category were never presented consecutively to minimize habituation effects. Each picture was presented for 6 s followed by an inter-stimulus interval which varied randomly between 9 and 13 s. During picture presentation the participant's ECG was recorded continuously.

Pictures were selected from a larger set in a pilot study that tested the suitability of the stimulus material with 39 healthy volunteers. Arousal and valence ratings for trauma versus generally threatening pictures did not differ significantly from each other. As intended, arousal ratings for trauma and generally threatening pictures were significantly higher than those for neutral pictures (*p*s < .001) and valence ratings were more negative (*p*s < .001).

Participants were informed that the purpose of the task was to measure bodily responses to pictures. They were instructed to sit down in a comfortable position and try to move as little as possible during the task. They were asked to look at each picture carefully.

### 2.3 Apparatus and physiological recording

Heart rate and respiration were recorded using the Varioport bio-signal recording device (Vitaport system, Becker Meditec). ECG electrodes were placed on the manubrium sterni and the left lower rib cage. The reference electrode was attached to the right lower rib cage and the ECG was recorded with a sampling rate of 256 Hz. To check for possible respiration artefacts (sighs or coughs), respiration was recorded continuously with a Pneumotrace II transducer which was attached around the participant's upper chest. Heart rate data from participants with frequent breathing artefacts or extra systoles were excluded from the analyses (see above).

### 2.4 Clinical interview and self-report measures

#### 2.4.1 PTSD diagnosis

PTSD was diagnosed with the *Structured Clinical Interview for DSM-IV* (SCID) (First et al., 1996). Interviewers were trained postgraduate psychologists. Interrater-reliability (determined from a random selection of 45 audiotapes of the interviews) for this sample was *κ* = 1.0.

#### 2.4.2 Self-reported PTSD symptoms

Participants completed the *Posttraumatic Stress Diagnostic Scale* (PDS) (Foa et al., 1997) at 1 and 6 month post-trauma. The PDS is a reliable, validated and widely used self-report measure of PTSD symptom severity. Participants rated how often they were bothered by each PTSD symptom as defined in the DSM-IV (American Psychiatric Association, 1994) on a scale ranging from 0 (*not at all or only one time*) to 3 (*5 or* more times a week/almost always) in the last month*.* The total severity score is the sum of all items. The internal consistency for the present sample was *α* = .93.

#### 2.4.3 Depressive symptoms

Severity of depressive symptoms was assessed with the Beck Depression Inventory (BDI) (Beck and Steer, 1987), a standardized self-report questionnaire with established reliability and validity. The internal consistency in the present sample was *α* = .92.

#### 2.4.4 Peritraumatic responses

Fear during the trauma was assessed with *Peritraumatic Emotions Questionnaire* (3 items, e.g. ‘terrified’, Evans et al., 2007; Halligan et al., 2003). Participants rated the extent to which they experienced these emotions during the trauma and until help arrived on a 5-point scale from 0 ‘not at all’ to 4 ‘very strongly’. Internal consistency was *α* = .86 in the present sample. Data-driven processing during the trauma was assessed with the *Cognitive Processing Questionnaire* (6 items, e.g. ‘It was just a stream of unconnected impressions following each other’). The questionnaire was developed in a series of studies (Halligan et al., 2002; 2003; Ehring et al., 2008), and showed good reliability and validity in predicting intrusive memories and PTSD. Internal consistency was *α* = .87 in the present sample.

#### 2.4.5 Dysfunctional coping responses

The *Safety Behaviors Questionnaire* assessed subtle avoidance behaviors and excessive precautions following trauma (13 items, e.g. ‘I check carefully whether doors/windows are locked*’*). It was developed over a series of studies (Dunmore et al., 1999; 2001; Ehring et al., 2008) and has shown good reliability and correlations with PTSD severity. Internal consistency was α = .90 in the present sample. Suppression of trauma memories was assessed with the thought suppression scale of the *Responses to Intrusions Questionnaire* (RIQ) (5 items; e.g. ‘I try to push them out of my mind’). The scale was developed in a series of studies (Clohessy and Ehlers, 1999; Ehlers et al., 1998; Halligan et al., 2003; Murray et al., 2002) and has shown good reliability and predictive validity. Internal consistency was *α* = .92 in the present sample.

#### 2.4.6 Appraisals

Overgeneralized appraisals of danger were assessed with the *Negative Thoughts about the World* subscale (5 items, e.g. ‘The world is a dangerous place’, ‘I have to be on guard all the time’) of the *Posttraumatic Cognitions Inventory* (PTCI) (Foa et al., 1999). Negative appraisals about the self were assessed with a short version of the *Negative Thoughts about the Self* subscale (5 items, e.g., ‘I am inadequate’ and ‘I can't rely on myself’). The PTCI has been shown to have good reliability, convergent validity and to discriminate between traumatized people with and without PTSD (Foa et al., 1999). Internal consistencies in the present sample were *α* = .76 and *α* = .87, respectively.

#### 2.4.7 Injury severity.

The Injury Severity Score (ISS) (Baker, 1974) is an anatomical scoring system which is based on the medical hospital notes and provides an overall score of the severity of injuries. A trained research nurse experienced in Emergency Medicine performed the ratings.

### 2.5 Procedure

Participants were recruited in two ways: where possible, participants (45%) were recruited in the Emergency Department on the day of their trauma. Participants who were admitted to the Emergency Room when no recruiter was present (55%) received an information sheet about the study and invitation letter 3 to 5 days after their trauma. Participants were assessed at 3 different time points: (1) upon recruitment (mean 12.8 days, SD = 14.9, median = 10), participants rated fear and data-driven processing during the trauma. ISS ratings were obtained on the basis of hospital notes. (2) Approximately 1 month after the incident (mean 40.5 days, SD = 13.1, median = 37), participants attended the research session that involved the *picture viewing task, SCID, PDS, and BDI.* The session also involved filling out other questionnaires that will be reported elsewhere. (3) Six months post-trauma (mean 184.0 days, SD = 42.0, median = 172), the *SCID* was conducted again over the telephone by the same interviewer and participants completed the PDS again.

### 2.6 Data reduction and statistical analyses

#### 2.6.1 Heart rate responses

HR data were pre-processed and analyzed with a software package developed by Gamer (2007). In a first step, R-waves were detected from the ECG data. R-R-intervals were then converted into HR (in beats per minute, bpm) and sampled on a real-time scale to obtain one weighted heart rate estimate for each second. The analysis followed guidelines for real-time analysis of cardiac activity (Velden and Wölk, 1987). For each picture, the pre-stimulus baseline HR was defined as the HR during the last second prior to picture onset (as in Bradley et al., 2001; Elsesser et al., 2004; Hamm et al., 1997). HR response was assessed as the relative change from baseline during the 6 s of stimulus presentation. For each second of stimulus presentation, the pre-stimulus baseline HR was subtracted from the HR during that second. The maximum of the HR responses for each second of stimulus presentation was used to index peak HR response.

#### 2.6.2 Statistical analyses

HR responses were analyzed with the General Linear Models (GLM) procedure in SPSS 15.0. The following factors were included in the GLM model: the between-group factor *diagnostic group* (PTSD vs. no PTSD at 1 month), the within-subject factor *picture type* (trauma, generally threatening and neutral), and the within-subject factor *second* (second one to six after stimulus onset). The Greenhouse–Geisser correction was used for the effects involving repeated measures.

According to Hypothesis 1, (greater HR responses to trauma-related pictures, but not for the two other picture types, in the PTSD group), we expected either a *diagnostic* *group* × *picture type* or a *diagnostic group* × *picture type* × *second* interaction, which was further analyzed by planned contrasts between the groups for each picture type.

Previous research has shown that healthy volunteers show greater HR deceleration in response to unpleasant than to neutral pictures (e.g., Lang et al., 1993; Bradley et al., 2001). Thus, we also expected a significant main effect of picture type, which was further analyzed with additional GLMs that tested which of the picture types differed significantly from each other.

As the PTSD and no PTSD groups differed in the proportion of participants who had experienced a MVA or assault, an exploratory GLM tested whether trauma type interacted with any of the factors specified in the GLM model. This was not the case. Similar analyses tested whether there were any interactions with sex or ethnicity (Caucasian vs. ethnic minority). As this was not the case either, the data are presented for the main 2 (diagnostic group) × 3 (picture type) × 6 (seconds) GLM.

Correlation analyses and logistic regression analyses were carried out using SPSS 15.0. The maximum (peak) HR response during the 6 s of presentation for trauma-related pictures was used in these analyses. PDS and BDI data were square root transformed to normalize distributions. An a priori significance level of α = .05 (two-tailed) was used for all statistical tests.

## 3 Results

### 3.1 Sample characteristics

Table 1 shows sample characteristics. Participants with and without PTSD at 1 month did not differ in age, injury severity, body mass index, or days since the trauma. Participants with PTSD were more likely to have been injured in an assault, to be female and from ethnic minorities than the no PTSD group. As expected, the PTSD group reported more severe PTSD and depressive symptoms than the no PTSD group, and reported more fear and data-driven processing during the trauma, more safety behaviors and thought suppression, and more negative appraisals.

### 3.2 Group differences in HR responses

**Fig. 1:**
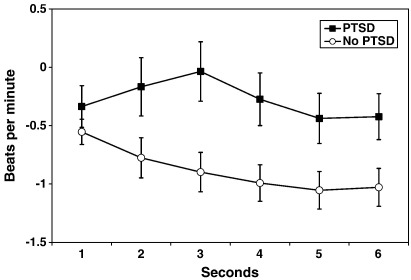
Mean heart rate (HR) responses to trauma-related pictures for the PTSD and no PTSD groups during the 6 s of stimulus presentation, relative to pre-stimulus baseline. The PTSD group showed greater HR responses (less HR deceleration) to the trauma-related pictures than the no PTSD group; but not to generally threatening or neutral pictures.

Pre-stimulus baseline heart rates did not differ between participants with (M = 68.2, SD = 11.0) and those without PTSD (M = 70.8, SD = 11.9), *p* > .16. In line with Hypothesis 1, the GLM showed a significant *diagnostic group* × *picture type* interaction, *F*(1,160) = 4.69, *p* = .010. Neither the *diagnostic group x picture type x second* interaction, *F*(1,160) = 1.66, *p* = .141, nor the main effect of *diagnostic group*, *F*(1,160) = 1.89, *p* = .171 were significant. The *diagnostic group* × *picture type* interaction was further analyzed by planned contrasts between the groups for each picture type. In line with the hypothesis, the PTSD group showed less pronounced HR deceleration (M = −0.28, SD = 1.56) to trauma pictures than the no PTSD group (M = −0.88, SD = 1.36), F(1,160) = 6.85, *p* = .010, *η*^2^ = .041. There were no such group differences for generally threatening, *F*(1,160) = 2.00, *p* = .160, *η*^2^ = .012, or neutral pictures, *F*(1,160) = 2.01, *p* = .158, *η*^2^ = .012. Fig. 1 shows the mean HR responses to the trauma-related pictures during the 6 s of stimulus presentation for the two groups.

**Fig. 2:**
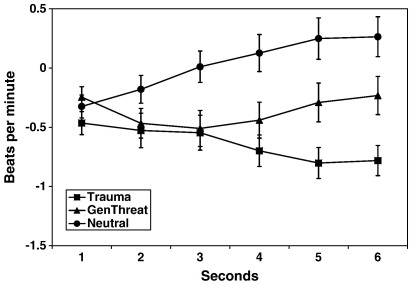
Mean heart rate responses to trauma-related, generally threatening and neutral pictures across both diagnostic groups for the 6 s of stimulus presentation.

In addition, the GLM showed a main effect for the factor *picture type*, *F*(1,160) = 6.72, *p* = .001) and a *picture type* × *second* interaction, *F*(1,160) = 6.91, *p* < .001. There were neither a main effect of *second, F*(1,160) = 0.98, *p* = .384, nor a *diagnostic group* × *second* interaction, *F*(1,160) = 1.13, *p* = .329. Further GLM showed that the main effect of *picture type* was due to significant differences in mean HR responses between trauma-related (M = − 0.64, SD = 1.47) and neutral pictures (M = 0.02, SD = 1.46), *F*(1,161) = 16.72, *p* < .001, *η*^2^ = .094, and between generally threatening (M = −0.36, SD = 1.50) and neutral pictures, *F*(1,161) = 6.22, *p* = .014, *η*^2^ = .037. There was a trend for a difference between trauma-related and generally threatening pictures, *F* (1,161) = 3.24, *p* = .071, *η*^2^ = .020. To further analyze the *picture type* x *second* interaction, separate GLM comparisons of the picture types were run for each of the 6 s of stimulus presentation. The results indicated that the differences between the picture types were only significant from second 3 onwards. The results are illustrated in Fig. 2.

### 3.3 Prediction of PTSD diagnosis by self-reports and HR responses

The mean peak HR response to trauma-related pictures was M = 0.78 (SD = 1.73) for the PTSD group and M = 0.05 (SD = 1.30) in the no PTSD group, *F*(1,160) = 9.561, *p* = .002. Of the PTSD group, 38% showed a HR acceleration of >1 bpm, compared to 13% of the no PTSD group *χ*^2^ (1, *N* = 162) = 12.778, *p* < .001, *OR* = 3.893, 95% *CI* = 1.807–8.388.

A logistic regression analysis showed that the peak HR response to the trauma-related pictures classified 57.4% of the participants correctly into the PTSD and no PTSD groups, with a sensitivity of 50% and a specificity of 63%, *χ*^2^(1,162) = 9.45, *p* = .002, Nagelkerke *R*^2^ = .076. A hierarchical logistic regression analysis tested how well a PTSD diagnosis at 1 month can be predicted from self-reports of PTSD symptoms (PDS) and depression (BDI) and peak HR response to trauma-related pictures. The PDS and BDI were entered in Step 1 and significantly predicted PTSD with an overall diagnostic efficiency of 80.6%, *χ*^2^(2, *N* = 160) = 93.04, *p* < .001, Nagelkerke *R*^2^ = .595. Peak HR response was entered in Step 2 and, in line with Hypothesis 2, significantly increased the accuracy of the prediction, *χ*^2^ (1, *N* = 160) = 4.06, *p* = .044, Nagelkerke *R*^2^ = .614.

### 3.4 Relationship between HR response and PTSD symptom severity

In keeping with the differences between the PTSD and no PTSD groups, peak HR response to trauma-related pictures correlated with PTSD symptom severity (PDS) at 1 month, *r* (161) = .22, *p* = .006. There was some support for Hypothesis 3. There was a trend for peak HR response to trauma-related pictures at 1 month to predict PTSD symptom severity (PDS) at 6 months, *r* (140) = .16, *p* = .058. Although the continuous peak HR response to trauma-related pictures at 1 month was not significantly related to PTSD diagnosis at 6 months, *F*(1,146) = 1.929, *p* = .167, *η*^2^ = .016, HR acceleration to trauma-related pictures of >1 bpm significantly predicted a diagnosis of PTSD at 6 months, *χ*^2^ (1, *N* = 148) = 5.441, *p* = .020, *OR* = 2.899, 95% *CI* = 1.156–7.270.

### 3.5 Relationship of HR response with peritraumatic responses, appraisals and coping

In line with Hypothesis 4, peak HR response to trauma-related pictures correlated with fear, *r*(159) = .22, *p* < .001, and data-driven processing during the trauma, r(160) = .29, *p* < .001. It also correlated with safety behaviors, *r*(155) = .30, *p* < .001, and effortful suppression of trauma memories, *r*(159) = .34, *p* < .001. It correlated with overgeneralized appraisals of danger, *r*(158) = .22, *p* < .001, but not with negative appraisals of the self, *r*(158) = .11, ns.

## 4 Discussion

The present study investigated whether HR response to standardized trauma-related pictures at 1 month after the trauma distinguishes between trauma survivors with and without acute PTSD. In line with Hypothesis 1, MVA and assault survivors with and without PTSD differed in their HR responses to trauma-related pictures, but not to generally threatening or neutral pictures.

The no PTSD group showed a HR deceleration to the trauma-related pictures that resembles the responses of healthy volunteers to unpleasant pictures observed in previous experiments and is generally interpreted as an orienting response (e.g., Lang et al., 1993; Bradley et al., 2001). The main effect of picture type indicating greater HR deceleration for trauma-related and generally threatening pictures compared to neutral pictures is also in line with the general literature on IAPS pictures (e.g., Lang et al., 1993; Bradley et al., 2001).

The PTSD group showed a different pattern of response to the trauma-related pictures from the no PTSD group: Their mean HR responses indicated a less pronounced cardiac deceleration during picture presentation compared to the no PTSD group. They also showed greater peak HR responses, which were in the accelerative range (M = +0.78), and a greater proportion showed pronounced HR acceleration greater than 1 bpm. Cardiac acceleration in response to threatening pictures has been previously observed in phobic or highly anxious patients (e.g., phobic patients watching pictures of their phobic object, Hamm et al., 1997; Sartory, 1983) and in PTSD patients watching idiosyncratic trauma reminders, Elsesser et al, 2004). It has been interpreted as a strong activation of the defensive system (Bradley et al., 1996).

These group differences for trauma-related pictures extend previous findings of heightened HR responses to *idiographic* trauma reminders in the initial months after trauma in civilian trauma survivors with PTSD (Blanchard et al., 1996; Elsesser et al., 2004). The present study demonstrated that trauma survivors with PTSD exhibit differential HR responses to *standardized* trauma-related pictures compared to those without PTSD as early as 1 month after the trauma. This result is in line with suggestions that PTSD is characterized by generalization of learned fear responses to stimuli that resemble the original traumatic situation (Keane et al., 1985; Foa et al., 1989). Generalization of conditioned fear responses is a well-established phenomenon. Animal studies have shown that conditioned emotional responses progressively generalize to more remote stimuli (Bouton and Moody, 2004; Delgado et al., 2006). The results suggest that by 1 month, the PTSD group's fear responses had generalized to general reminders of the trauma.

In line with Hypothesis 2, the present study showed that HR responses to trauma-related pictures at 1 month predicted acute PTSD over and above what could be predicted from self-reports of PTSD symptoms and depression. This result may have some practical relevance. While the proportion of additional explained variance was small, HR responses may provide useful information in situations where there is reason to believe that people may overreport or underreport symptoms. On its own, however, HR response did not have sufficient sensitivity (50%) and specificity (63%) to be useful for diagnostic purposes and was lower than that reported for self-report screening instruments for PTSD, many of which exceed minimum sensitivities and specificities of 75% (Brewin, 2005; Ehring et al., 2007). The sensitivity and specificity of HR responses were lower than those reported in Pole's meta-analysis of studies of chronic PTSD. One possible explanation is that as PTSD becomes chronic, the generalization of fear responses increases further. This hypothesis could be investigated in future studies by assessing HR responses to trauma reminders repeatedly in the course of the disorder. In line with results on self-reported physiological responses to trauma reminders (Ehlers et al., 1998; Ehring et al., 2008), the specificity of HR responses in predicting PTSD (63%) was greater than its sensitivity (50%).

The moderate sensitivity of 50% indicates that there may be several pathways to chronic PTSD, and generalized fear responses may only be one of them. This pattern of findings is in line with theories of PTSD that suggest several maintenance factors. For example, cognitive models of PTSD distinguish between appraisals about external threat and those about the self (e.g. Ehlers and Clark, 2000). Both types of appraisals have been shown to predict PTSD (e.g., Dunmore et al., 2001; Halligan et al., 2003; Ehring et al., 2008), and people with PTSD differ in which type is more relevant to them. HR response to trauma-related pictures was only expected to correlate with appraisals of external threat, and the results were in line with this hypothesis. HR response was related to overgeneralized appraisals of external danger, but not to negative appraisals of the self. Thus, HR responses may be one way of distinguishing between different subtypes of PTSD, as Wilhelm and Roth (2001) suggested. HR response to trauma-related pictures appears to identify those PTSD patients who resemble patients with phobia in that they are afraid of external danger, take excessive precautions and show phobic avoidance.

The results for the prospective prediction of PTSD symptoms (Hypothesis 3) were mixed. HR response to trauma reminders at 1 month tended to predict PTSD severity at 6 months. While the continuous HR response score did not significantly predict PTSD diagnosis at 6 months, HR acceleration to trauma-related pictures >1 bpm predicted PTSD diagnosis at follow-up. This result indicates that HR acceleration may be of greater clinical significance than the continuous peak response score in identifying people at risk of chronic PTSD. The findings extend previous longitudinal studies showing that HR acceleration to individualized trauma reminders predicts chronicity of PTSD symptoms (Blanchard et al., 1996; Elsesser et al., 2005).

The correlations between HR response to trauma-related pictures and fear during the trauma are in line with associative learning models of PTSD (Keane et al., 1985; Foa et al., 1989). Fear during the trauma predicted greater HR response to trauma-related pictures at 1 month follow-up. High fear during trauma may indicate a strong activation of the sympathetic nervous system and thus lead to stronger conditioning of emotional responses, which in turn may increase the risk for PTSD. The finding that data-driven processing during the trauma also predicted HR response to trauma-related pictures lends preliminary support to the suggestion that engaging in perceptual processing during trauma promotes associative learning (Ehlers et al., 2004).

The present study has strengths and limitations. Among its strengths are the large sample size and the early assessments of early symptoms and HR responses. Furthermore, trauma survivors were diagnosed with reliable structured clinical interviews. A limitation was that arousal and valence ratings for the pictures were not taken in the main study so that possible differences between the PTSD and no PTSD groups could not be assessed. In addition, it would have been interesting to examine the concordance between self-reported arousal and valence with physiological responses to trauma reminders. The lack of assessment of a baseline HR before the experiment is a further limitation.

In conclusion, we found that at 1 month after the trauma, trauma survivors with acute PTSD showed greater HR responses to standardized visual trauma reminders than those without PTSD. HR responses predicted PTSD diagnosis over and above what could be predicted from self-reported PTSD symptoms and depression. The pattern of correlations suggested that HR responses characterize a subtype of PTSD that shows overgeneralized fear responses. The results are in line with Orr and Roth's (2000) suggestion that physiological measures may help identify people with PTSD, and Wilhelm and Roth's (2001) suggestion that physiological measures that map onto core symptoms may be relevant in identifying subgroups of anxiety disorders such as PTSD.

## Figures and Tables

**Table:** 

Picture	Assault-related (for assault survivors)	MVA-related (for MVA survivors)	Generally threatening (all participants)	Neutral (all participants)
1	Man attacking woman	Person in crashed car with airbag	Building collapsing	Basket
	IAPS No: 6312		IAPS No: 9470	IAPS No: 7010
2	Bleeding head	Bleeding head	Man in chemical suit	Book
				IAPS No: 7090
3	Bloody hands	Broken car window	Brown rat	Dustbin
4	Pedestrian in dark street	Cyclist being cut off by car	Drowning man	Glass
5	Gang attacking men	Swerving motorcyclist	Flooded houses	Keyboard
6	Hand with knife	Smashed bike and car	Man falling from roof	Lamp
	IAPS No: 6300			IAPS No: 7175
7	Man covered in blood	Injured person trolley	Tank	Plug
	IAPS No: 3550			
8	Subway	Man off bike	Fire fighter rescuing woman	Rolling pin
			IAPS No: 9921	IAPS No: 7000
9	Man running away with bag	Man being rescued from car	Plane crash	Toothpaste
			IAPS No: 9050	
10	Knife on throat	Ambulance and stretcher	Woman holding on to from roof	TV
	IAPS No: 6550			
11	Skinheads with bats	Pedestrian crossing road		
12	Man being kicked	Car crashed into tree		
13	Man attacking with broken bottle	Collision of car and motorbike		
14	Policeman with truncheon	Crashed bike and car		
	IAPS No: 2682			
15	Woman being watched on street	Woman in crashed car		

Legend: MVA: motor vehicle accident; IAPS: International Affective Picture System

**Table 1:** Sample and Trauma Characteristics (N and percentage, or Mean and SD).

Variables and Range	PTSD Group (N = 66)	No PTSD Group (N = 96)	Statistics for Group Difference	
Sex			χ^2^ (1, N = 162) = 5.64	p = .018
Female	29 (43.9%)	25 (26.0%)		
Male	37 (56.1%)	71 (74.0%)		

Age (in years) (18 – 61)	31.9 (10.9)	33.3 (11.6)	t(160) = 0.79	p = .433

Type of trauma			χ^2^(2, N = 162) = 11.13	p = .001
Assault	38 (57.6%)	30 (31.3%)		
Motor vehicle accident	28 (42.4%)	66 (68.8%)		

Ethnic origin			χ^2^ (2, N = 162) = 18.37	p = .001
Caucasian	22 (33.3%)	64 (66.7%)		
Black	27 (40.9%)	23 (24.0%)		
Other (e.g. mixed)	17 (25.8%)	9 ( 9.4%)		

Level of Education			χ^2^ (3, N = 160) = 5.13	p = .162
No exams	12 (18.8%)	12 (12.5%)		
GCSE (11 yrs of school)	32 (50.0%)	38 (39.6%)		
A level (13 yrs of school)	10 (15.6%)	18 (18.8%)		
Bachelor degree or above	10 (15.6%)	28 (29.2%)		

Injury severity score (ISS)	2.13 (2.70)	1.91 (1.66)	t(140) = 0.60	p = .552

Body mass index	24.1 (4.1)	25.3 (4.3)	t(136) = 1.72	p = .087

Days between trauma and picture viewing task	42.2 (13.8)	39.5 (12.6)	t(159) = 1.32	p = .190

PDS score (1 month) (0–51)	26.1 (9.7)	10.1 (8.2)	t(159) = 11.46	p < .001

PDS score (6 month) (0–51)	18.7 (12.2)	6.1 (6.6)	t(138) = 7.54	p < .001

BDI score (1 month) (0–63)	17.5 (10.6)	5.6 (5.9)	t(158) = 9.29	p < .001

Data-driven processing (during trauma) (0–4)	2.45 (1.01)	1.58 (1.01)	t(158) = 5.35	p < .001

Fear (during trauma) (0–4)	2.64 (1.21)	2.04 (1.25)	t(157) = 3.00	p < .001

Safety behaviors (1 month) (0–3)	1.95 (0.51)	1.32 (0.66)	t(153) = 6.31	p < .001

Thought suppression (1 month) (0–3)	1.80 (0.68)	1.06 (0.85)	t(157) = 6.01	p < .001

Overgeneralized appraisals about dangerousness of the world (1–7)	5.17 (1.26)	3.28 (1.34)	t(156) = 7.33	p < .001

Negative appraisals of the self (1–7)	2.62 (1.41)	1.41 (0.65)	t(156) = 8.44	p < .001
